# Multilayer bonding of A1N30H foils to A1050 plates using cold spot forge welding

**DOI:** 10.1016/j.heliyon.2023.e23103

**Published:** 2023-11-29

**Authors:** Hideki Yamagishi, Yasukazu Hisada, Takuya Otsubo, Noburo Omori

**Affiliations:** a Toyama Industrial Technology Research and Development Center, Takaoka, 933-0981, Japan; b Dengensya Toa Co. Ltd., Kawasaki, 214-8588, Japan

**Keywords:** Aluminum foil, Cold spot forge-welding, Multilayer, Solid-phase bonding, Tab electrode

## Abstract

The cold spot forge-welding method, recently developed to achieve high-productivity and high-strength dissimilar material joining, was applied to multiple solid-phase joining of aluminum (Al) foils simulating tab-lead electrodes. Fifty Al foils of 12-μm thickness were sandwiched between 0.5 and 0.8 mm A1050 Al alloy plates and pressurized with 6-mm diameter punch for 1 s. The effect of bonding temperature (330–420 °C) and the reduction ratio (*R*, 1.4–3) on the tensile shear load of the joint was investigated. A lower *R* value at a higher bonding temperature resulted in base metal fracture (*i.e.*, plug fracture) of the Al alloy plate. The maximum load reached 410 N using a reduction ratio of higher than 2.1 and bonding temperature of 420 °C. The processed foils were properly stretched in the plane without breakage, and a total of 51 sound layered bonded interfaces were formed. The results also confirmed that the oxide film became more rarefied with increasing *R*. These results are expected to be applicable to high-throughput, high-reliability bonding of secondary battery electrodes.

## Highlights

• Multilayer A1N30H foils, sandwiched between A1050 plates were bonded by cold spot forge welding.
• The foils formed 51 sound solid-phase bonded interfaces with no breakage.
• Shear tensile test showed Al plate fracture, not foil.
• Oxygen concentration at the bonded interface decreases with increasing reduction ratio.

## Graphical abstract

**Figure:**
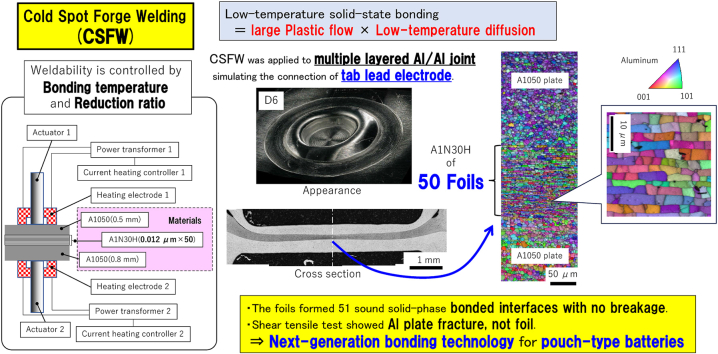

## 1 Introduction

The electrification of mobility requires high-quality, efficient connection technology for various electrical components such as motors, rechargeable batteries [1,2], and harnesses. For example, pouch-type lithium-ion battery cells require direct multiple connections of foil-like tab leads from each cell. This usually requires solid-phase bonding to avoid intermetallic compound (IMC) problems with fusion welding and contamination problems such as spatter. Ultrasonic welding (UW) is one of the main processes used [[1], [2], [3]]; however, the productivity and quality outcome of this method are limited by horn wear and unbonded regions caused by ultrasonic energy attenuation at the bottom interfaces [4]. In terms of dissimilar lap joining of aluminum (Al) and copper (Cu) thin plates, M. P. Satpathy et al. proposed a three-dimensional thermo-mechanical element model and demonstrated with experiments the thermo-softening phenomena in the UW process [5]. M. D. Leon and H. S. Shin reviewed and discussed modeling and simulation using the finite element method to predict physical phenomena in dynamic UW processes, addressing important issues related to process properties and key concerns about joint quality [6]. J. Li et al. conducted qualitative and quantitative investigations of plastic deformation, microstructural changes, and related mechanical properties in UW and showed that the mechanical properties of joints are strongly influenced by plastic deformation and microstructural changes [7]. Among solid-phase bonding methods, UW has been one of the most actively studied for battery use, but there are still various problems to be solved. Thus, a point solid-phase bonding method with higher productivity and quality performance is desired.

A next-generation solid-phase welding method was recently developed [[8], [9], [10]]. To distinguish it from the conventional forge-welding (FW) method, it is called cold forge-welding (CFW); in spot form, it is referred to as cold spot forge-welding (CSFW) [11]. CFW is a low-temperature process in which the ratio of the bonding temperature (*T*) to the melting temperature of the metal is 0.3–0.7. In CFW, diffusion bonding of metals occurs directly over a short period, without the use of flux or insert materials. This approach is virtually IMC-free [12] and can render a reaction layer (RL) that is harmless for dissimilar metals. Control over the metallurgical RL at the interface, which is the bonding mechanism, is carried out by adjusting the reduction ratio (*R*) and bonding temperature. This allows bonding and forming to be achieved simultaneously within a short period, making CFW a highly productive multi-material technology. Excellent bonding properties have been reported for spot forms of 1-mm-thick strips of iron/Al [8,12,13], Al/Al [13], Cu/Al [11], nickel/Al [14], and titanium/Al [15].

In this study, a CSFW method was applied to Al foil multi-layered bonding to investigate the effects of bonding temperature and *R* on the tensile shear load of the joint. The results also confirmed that the oxide film became more rarefied with increasing *R* at the bonded interface (BI), which had not been realized in previous studies.

## 2 Experimental

**Fig. 1:**
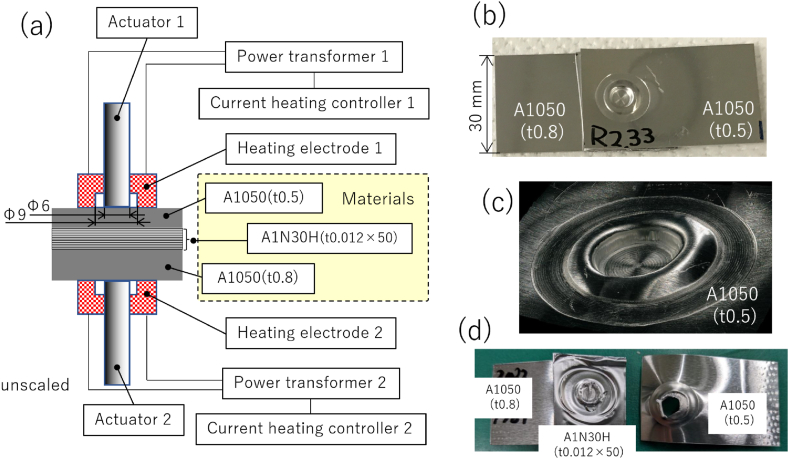
(a) Schematic illustration of the cold spot forge-welding system. (b) An example of the forged joint. (c) A digital microscope image of the bonded area. (d) Example of the cold forged joint with a plug fracture in tensile testing (0.5-mm-thick A1050 base metal fracture).

Al alloy (A1050) plates (dimensions: 30 × 50 × 0.5 or 0.8 mm^3^) and Al (A1N30H) foil (dimensions: 30 × 30 × 0.012 mm^3^) were used as the test materials. The chemical compositions are listed in Table 1. Fifty sheets of Al foil, laminated and sandwiched between 0.5- and 0.8-mm-thick top and bottom Al plates, were joined using a prototype CSFW machine (Fig. 1a) [16]. To control the bonding temperature, the specimens were preheated indirectly via a heating electrode made of SUS304, rather than directly energized. Bonding temperatures ranging from 330 °C to 420 °C were applied. Temperature was measured by placing a 0.5 mm diameter K-type thermocouple across the junction interface. Bonding tests were conducted after preheat holding times determined to stabilize at each target bonding temperature. The center of the sandwich was pressurized for approximately 1 s using an SKD61 rod with a 6-mm-diameter flat surface. Pressurization was performed using an air cylinder with an axial force of 2 kN, and the stroke was mechanically displacement-controlled with a stopper. In this experiment, the lower rod was fixed and pressure was applied from the upper rod.

**Table 1:** Chemical composition of the materials (mass %).

Material (JIS H 4000)	Si	Fe	Si + Fe	Cu	Mn	Mg	Zn	Ti	V	Al
A1050 (t0.5 mm)	0.10	0.28	–	0.01	Trace	Trace	Trace	0.03	Trace	99.57
A1050 (t0.8 mm)	0.07	0.32	–	0.01	Trace	Trace	Trace	0.02	0.02	99.55
A1N30H	–	–	0.62	0.02	Trace	Trace	Trace	–	–	99.36

*R* is the ratio of the specimen wall thickness before and after bonding, defined as the before-processing thickness (1.9 mm) divided by the after-processing thickness at the bonded center point. For the tests conducted, *R* ranged from 1.4 to 3.0. Fig. 1b and c shows photographs of the joint at *R* = 2.3, and a magnified view of the joint using a digital microscope, respectively. The material was forced out around the joint like a floating ring to obtain the specified *R*. The joints were subjected to a tensile shear test to investigate the maximum load (*F*) and the failure mode. The photograph in Fig. 1d shows an example of a base metal (BM) fracture (*i.e.*, plug fracture) in the joint after tensile testing. Cross-sectional microstructural observation was performed using optical microscopy, electron backscattering diffraction (EBSD), and field-emission scanning electron microscopy. To verify the effect of BI cleanliness with increasing *R*, changes in the oxygen (O) distribution at the BI were evaluated using electron probe microanalysis (EPMA). Additional detail of the BI was observed by transmission electron microscopy (TEM) based on a focused ion beam (FIB) sampling method.

## 3 Results and discussion

**Fig. 2:**
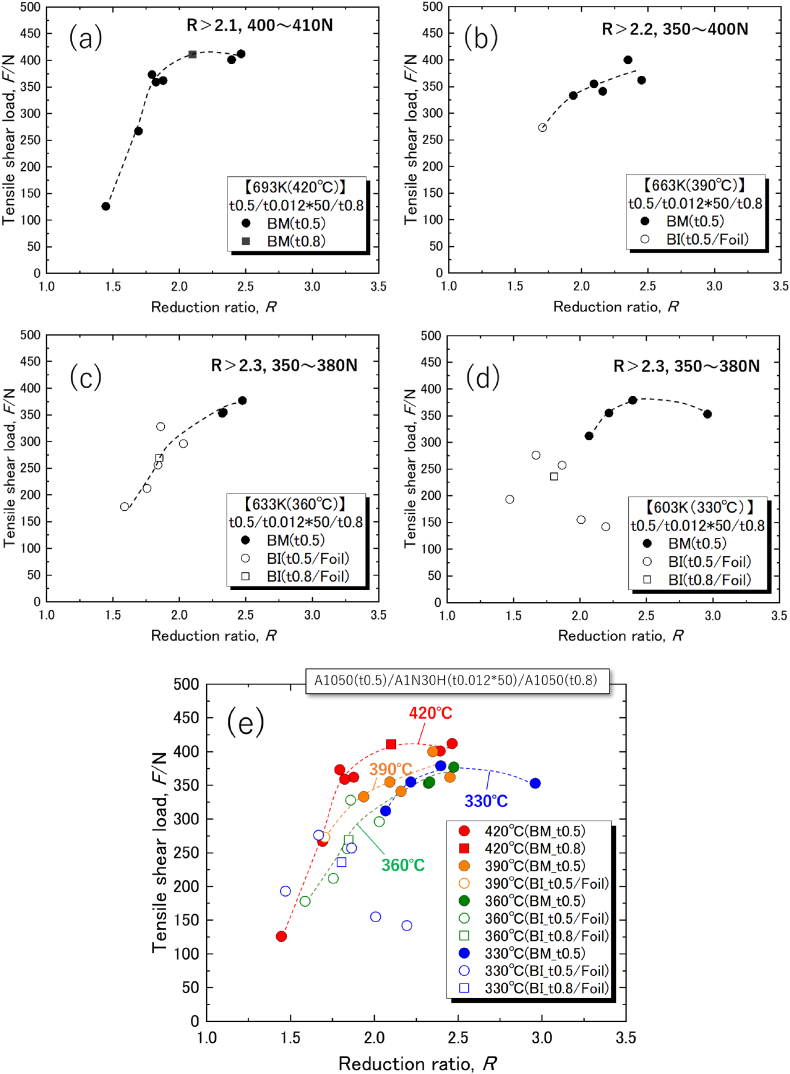
Effect of the reduction ratio on tensile shear load of joints at bonding temperatures of (a) 420 °C, (b) 390 °C, (c) 360 °C, and (d) 330 °C. (e) Overlay of all data.

Fig. 2a–d shows the effect of *R* on *F* at bonding temperatures of 420 °C, 390 °C, 360 °C, and 330 °C, respectively. Fig. 2e shows an overlay of the data in which dark-shaded areas indicate a BM fracture and white areas indicate a BI fracture; the fracture location is noted in parentheses. Joint strength increased significantly with *R* at all temperatures, and the failure mode transitioned to BM fracture. At higher bonding temperatures, BM fracture occurred at lower *R* values. Thus, high *R* values enable sound bonding at lower temperatures. At 420 °C, the highest bonding temperature, *F* exceeded 400 N, resulting in an *R* value of 2∼2.5, which held the highest joint strength. Conversely, at 330 °C, the lowest bonding temperature, the BI mode before the transition to BM fracture showed a large variation and poor controllability based on *R*; this case was exemplified by a diffusion bonding mechanism at the interface, and its controllability was usually governed by the fundamental parameters, bonding temperature and *R*, in the appropriate range [11,15]. At 330 °C and *R* < 2.2, the effect of the contaminated layer, which served as a diffusion barrier layer, became more dominant. Although *R* is measured at the center of the joint diameter for convenience, in reality, plastic flow is not uniform within the plane, especially at low temperatures and low *R* values. It is assumed that the quality of the joint surface is unstable within the plane, and that the low diffusion driving force causes variations in the diffusion reaction and its area with metallurgical joining power.

**Fig. 3:**
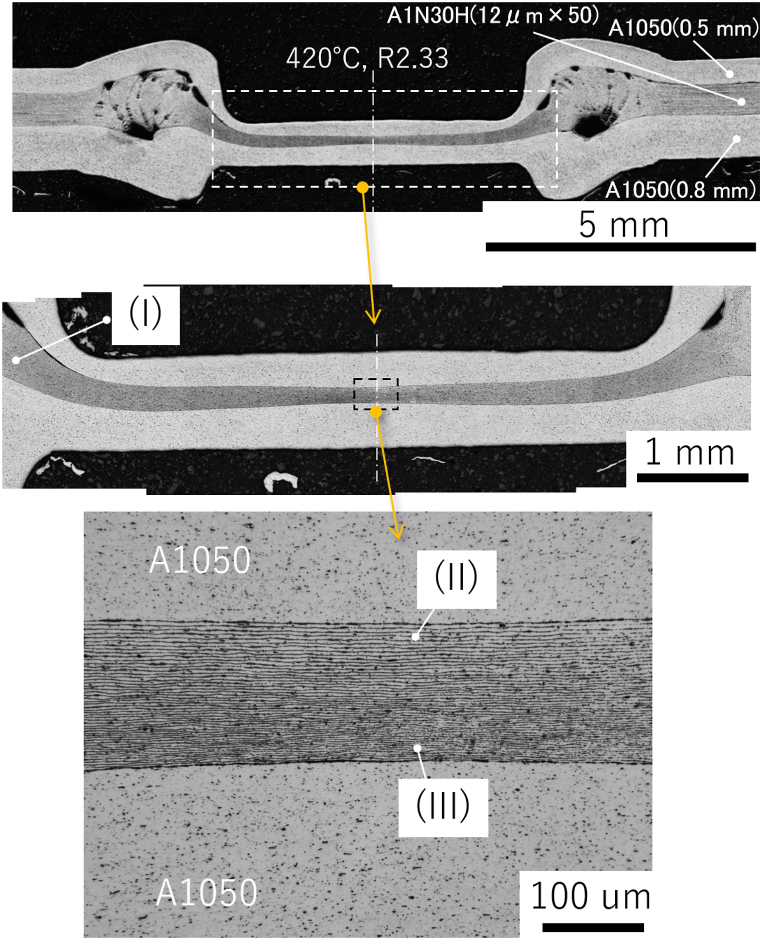
Optical microscopy of the cross-section at the bonded interface for a joint at 420 °C bonding temperature and reduction ratio (*R*) of 2.33.

**Fig. 4:**
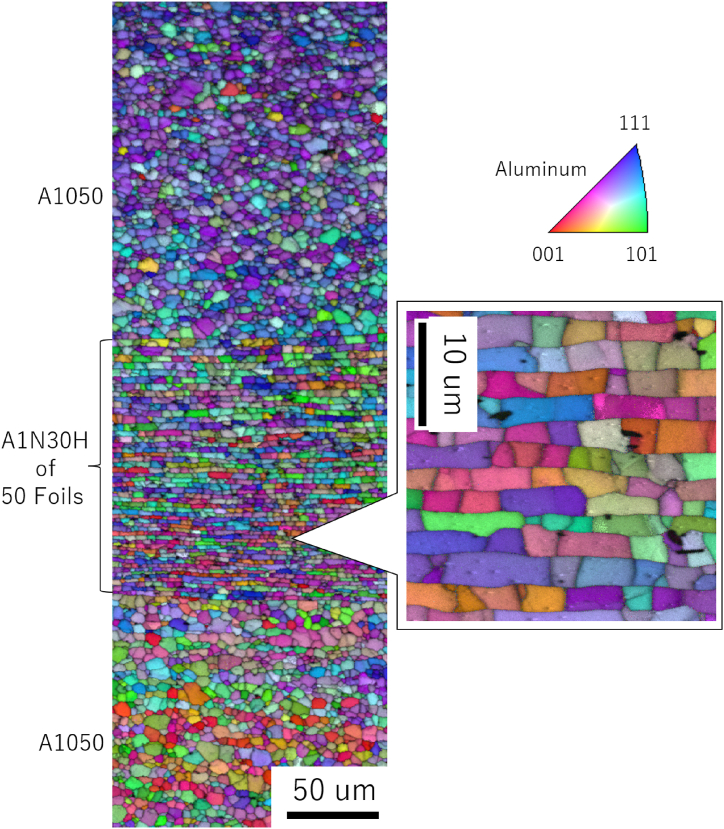
Inverse pole figure with an image quality map of the cross-section at the bonded interface for Fig. 3.

**Fig. 5:**
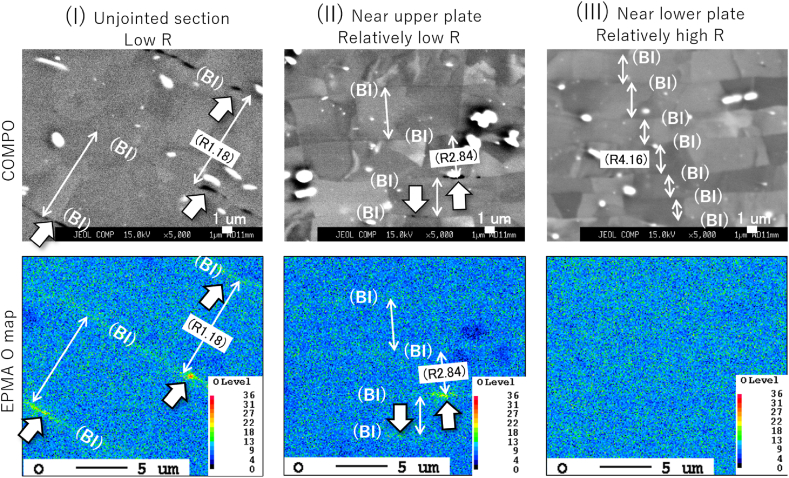
Reflection electron images (upper panel) and electron microanalysis elemental maps (lower panel) at positions I, II, and III for Fig. 3.

**Fig. 6:**
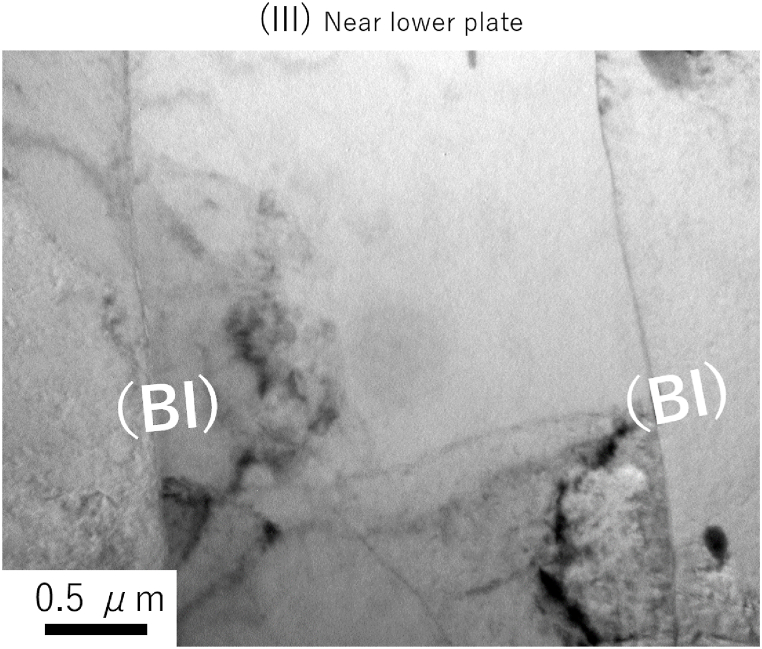
Transmission electron microscopy bright-field image for the III region in Fig. 5.

**Fig. 7:**
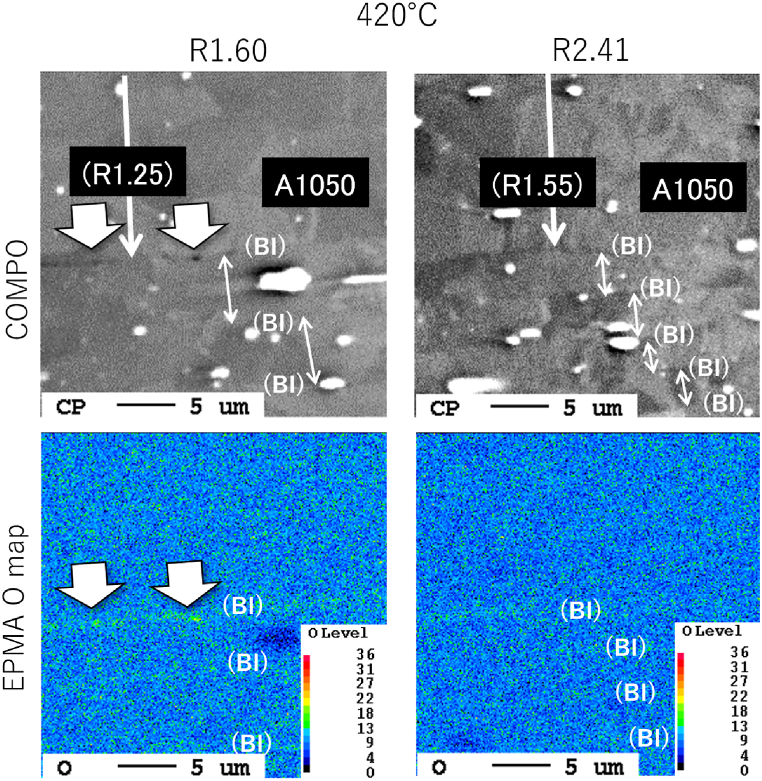
Reflection electron images (upper panel) and electron microanalysis elemental maps (lower panel) at the bonded interface between the upper plate and the foil for *R* of 1.60 and 2.41.

Fig. 3 shows a cross-sectional macroimage of the center of the joint formed at 420 °C; the *R* value was 2.33 and this joint exhibited the highest joint strength. The specimen cross-section was etched with a 2 % sodium hydroxide solution. The materials were forced out around the joint, forming a floating ring. This ensured bonding interface stretching (*i.e*., interface cleaning) of the joint. Note that there is a gap between the plate and the foil in the ring area, which is contaminated by polishing and etch-pits, but the interface between the plate and the foil is smooth and produces little effect of mechanical bonding. The cross-sectional thickness was roughly constant throughout the BI; however, the foil tended to be thinner at the bonded center. Given that *R* affects the efficiency of the diffusion reaction, these results imply that the local bond strength depends on the local interface location. There is no problem in practical control criteria using a plug fracture as the standard. However, if adjustments are to be made, such as a constant *R* within the bonding plane, a rod shape with crowning may be necessary. Fig. 4 shows an EBSD image with an inverse pole figure map of Fig. 3. The foils were free of breakage and maintained a layered structure that comprised 51 BIs. The *R* of the foil was about 2.8 on the pressurized side and about 4.2 on the fixed side, indicating a relatively low *R* value on the pressurized side. Fig. 5 shows the reflection electron images and EPMA maps of O (*i.e.*, oxide layer) at the positions shown in Fig. 3. Three positions (I–III) were analyzed in more detail: the low-*R* region (*R* = 1.18) outside the bonding area (position I); the relatively low-*R* region (*R* = 2.84) on the pressurized side near the BI center (position II); and the relatively high-*R* region (*R* = 4.16) on the fixed side (position III). The increase in *R* showed that the O intensity of BI was almost unidentifiable by EPMA at position III; specifically, the effect of the diffusion obstacle layer appeared to be reduced, resulting in a cleaner state that allowed diffusion even at lower temperatures. It should be noted that the introduction of *R* did not discharge the contaminant layer outside the bonding area but rather stretched and broke up the layer locally *in situ* to a level that was undetectable by EPMA. Fig. 6 shows a TEM bright-field image of the (III) region. Since the materials are pure Al and do not form IMC, we cannot confirm a clear RL as in the case of dissimilar materials bonding, but the FIB sampling shows that the interface is not exfoliated and that is clearly diffusion bonded. A similar Al/Al BI is shown in ref. 13. Finally, for the BI between the upper plate and the foil, which was the main location for BI fracture, reflection electron images and element maps are shown in Fig. 7, as in Fig. 5. The *R* of 1.60 and 2.41 for the bonding temperature of 420 °C are compared. Here, the *R* for upper plates is 1.25 and 1.55, respectively. As *R* increases, the cleanliness improves and voids disappear, resulting in a sound solid-phase BI. The CSFW method can be applied to tab leads, and the joint quality of the interface can be controlled by the *R* value and the bonding temperature.

## 4 Conclusions

A CSFW method was applied to multi-layered Al/Al solid-phase bonding simulating tab-lead electrodes. Fifty A1N30H foils were sandwiched between A1050 plates, and high-strength bonding was achieved with a BM fracture of the Al plates under pressurization for 1 s. The maximum tensile shear load of the joint reached 410 N at a bonding temperature of 420 °C and an *R* value of 2.1 or higher. The foils were free of breaks and maintained a layered relationship, with the joint consisting of 51 sound solid-phase BIs. The improved cleanliness of the BI with *R* was visualized by EPMA O map analysis. High *R* makes the oxide film, which is an obstacle layer in diffusion, less effective; this explains the transition to a BM fracture even at lower temperatures with higher *R* values. The results help clarify bonding mechanisms, and are expected to have application in tab-lead electrode bonding as a high-speed, high-strength solid-phase bonding process.

## Data availability statement

Data associated with the study has not been deposited into a publicly available repository and data will be made available on request.

## CRediT authorship contribution statement

**Hideki Yamagishi:** Conceptualization, Data curation, Investigation, Methodology, Writing – original draft, Writing – review & editing. **Yasukazu Hisada:** Conceptualization, Investigation, Methodology. **Takuya Otsubo:** Data curation, Investigation, Methodology. **Noburo Omori:** Conceptualization, Project administration, Supervision.

## Declaration of competing interest

The authors declare that they have no known competing financial interests or personal relationships that could have appeared to influence the work reported in this paper.
